# Trap Design for the Brown Recluse Spider, *Loxosceles reclusa*

**DOI:** 10.1673/031.013.5701

**Published:** 2013-06-20

**Authors:** Jennifer Parks, William V. Stoecker, Robert L. Paige

**Affiliations:** 1 SpiderTek, LLC, 10101 Stoltz Dr. Rolla, Missouri; 2 Department of Dermatology, University of Missouri Health Science Center, Columbia, MO; 3 Department of Mathematics and Statistics, Missouri University of Science and Technology

**Keywords:** arachnid, glue-trap

## Abstract

While there are limited options for chemical-free Arachnid pest control, glue-traps are one suitable alternative to pesticides. The effectiveness of several three-dimensional glue-trap shapes for trapping the brown recluse spider, *Loxosceles reclusa* Gertsch and Mulaik (Araneae: Sicariidae), was investigated using four novel glue-trap shape designs, which were compared to an existing design currently on the market. These four novel and one standard shape designs were tested using pairwise comparisons. The most preferred trap design was a flat glue-trap with no covering. Although this type of trap was most efficient for capturing *L. reclusa*, it can pose risks in homes with children and pets for obvious reasons. Among the traps with coverings, the vertical strut trap was most preferred by the spiders, and should perhaps be the trap of choice for homeowners with children and pets.

## Introduction

The brown recluse spider, *Loxosceles reclusa* Gertsch and Mulaik (Araneae: Sicariidae), is a common household pest in the Midwestern United States. Many homeowners, however, are deterred from using chemical pesticides due to possible health risks and environmental side effects. A viable non-chemical alternative is a glue-trap. Glue-traps have been sold commercially for capture of not only arachnids but also flying insects, rodents, and reptiles. Glue-traps have also been used for estimating the population of beetle infestations (e.g., Hagstrum et al. 1994). In addition, glue-traps have been used to estimate brown recluse populations inside residential housing (Vetter et al. 2002). A search of the existing literature reveals no studies that compare spider trap designs, even though spider populations have been successfully estimated with glue-traps (Sandidge et al. 2005).

Although a few different-sized traps are currently on the market, the basic form is a boxlike or rectangular shape. Few commercial traps are not shaped with right angles, like a box. They do not employ angles less than 90 degrees, even though at least one *Loxosceles* species prefers refuges that offer acute angles (Stropa 2010). In our study, a total of four novel trap shape designs and one popular glue trap already on the market were tested to determine if one (or more) of the new designs were more likely to catch brown recluse spiders than the existing design.

*L. reclusa* prefer dark, undisturbed places, although they do wander in search of mates and prey items (personal observation). Although reclusive and shy, *L. reclusa* have shown a preference for certain surfaces, such as cardboard, newspaper, and lumber (personal observation), and other *Loxosceles* species have shown similar preferences (Fischer et al. 2005). Of these choices, cardboard is the most practical and most inexpensive choice for trap construction. It was hypothesized that glue traps employing cardboard would be suitable for attracting and trapping these spiders. The motivation of this study was to determine the optimal three-dimensional shape(s) of cardboard traps for catching brown recluse spiders.

## Materials and Methods

**Figure 1.**
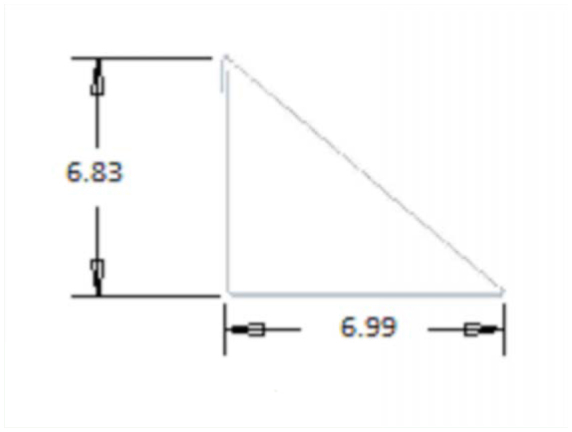
Side measurements of traps. High quality figures are available online.

**Figure 2.**
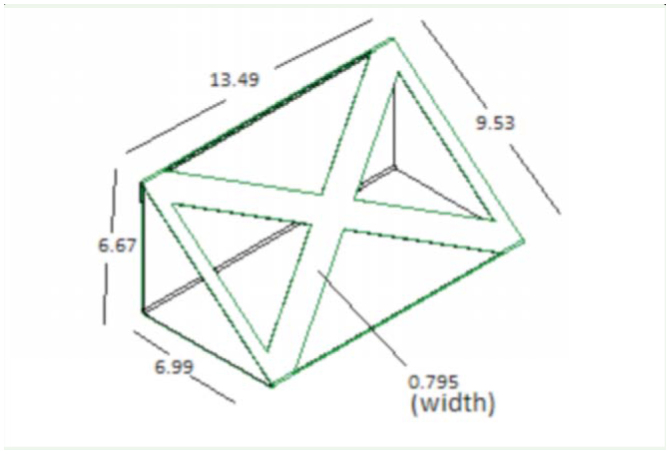
X-shaped strut design. High quality figures are available online.

**Figure 3.**
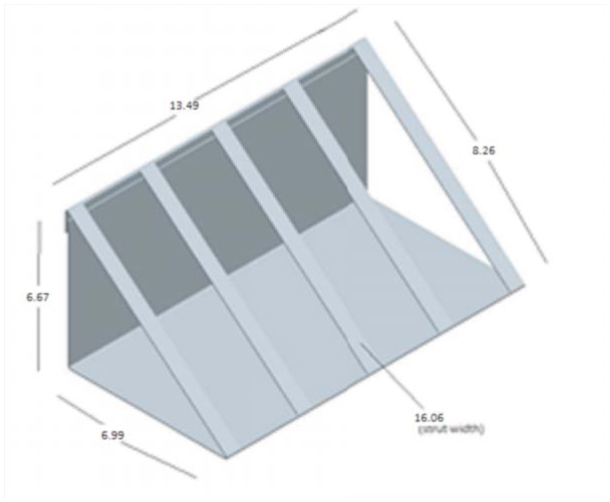
Vertical strut design. High quality figures are available online.

**Figure 4.**
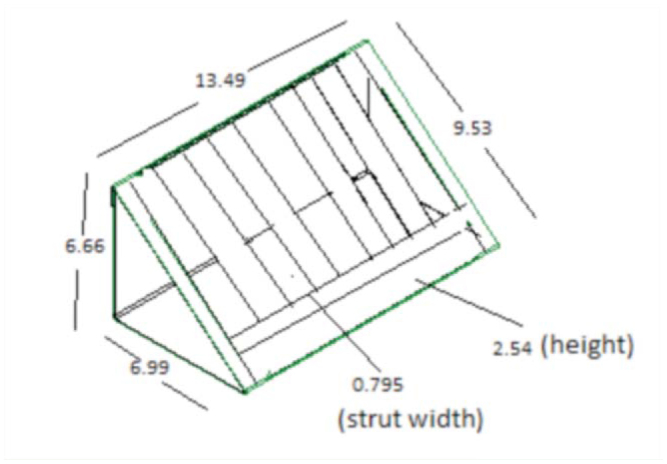
Vertical struts with horizontal bar design. High quality figures are available online.

**Figure 5.**
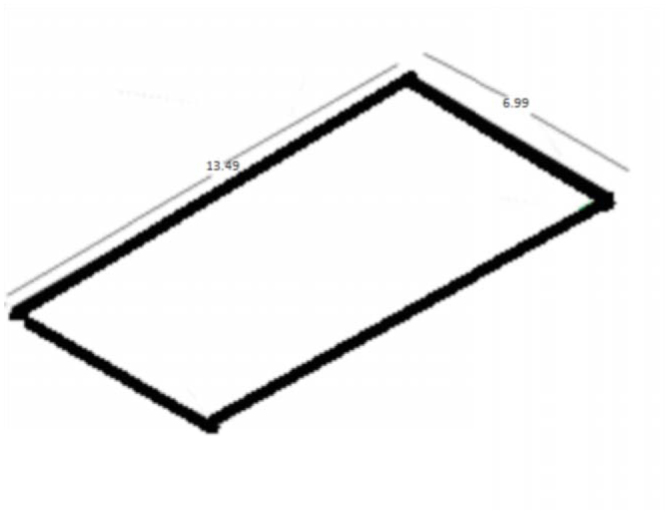
Flat trap design. High quality figures are available online.

**Figure 6.**
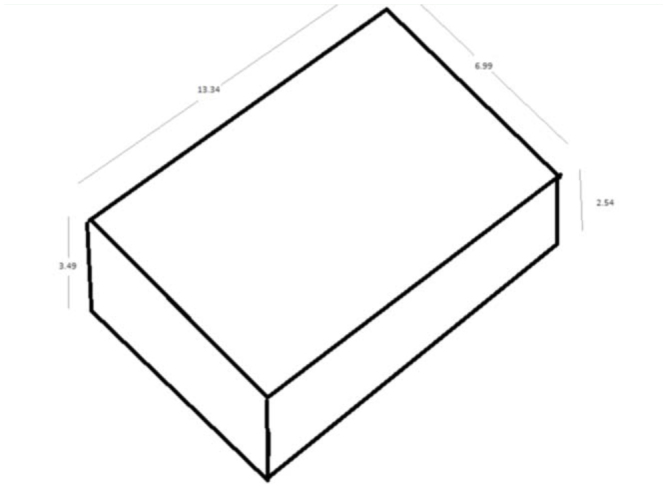
Control trap design. High quality figures are available online.

All *L. reclusa* used in this study were caught in central or south-central Missouri, USA. While in the laboratory, they were fed a diet consisting of domestic house crickets (*Achetes domesticus*) and various species of shorthorned grasshoppers. A mixture of adult and juveniles spiders were used. Glue-trap designs were made using modified Catchmaster gluetraps (www.catchmaster.com) cut into 6.67 × 13.49 cm rectangles and laser-produced cardboard cutouts from The Center for Rapid Product Realization at Western Carolina University. The experimental roofed traps used 0.03 in. non-corrugated chipboard pad cardboard (Uline, www.uline.com) laser cut to the specifications shown in Figures 1–4. There were a total of five trap designs: flat (6.67 × 13.49 cm rectangle with no cardboard attached), X-shaped struts, vertical struts, horizontal bar with vertical struts, and regular Catchmaster traps, which were used as the control (Figures 1–6). The base of the trap was the same (6.67 × 13.49 cm rectangle) in each of the five designs.

For a paired comparison of traps, two spiders of the same gender and/or age group (males with males, females with females, juveniles with juveniles) were placed into a plastic bin measuring 30.48 × 45.72 × 30.48 cm and left to acclimate for approximately 12 hours. At that point, two traps of different designs were placed in the bin, one on either end, about 2.54 cm from the wall. Spiders were left for another 12 hours, and at the conclusion of that period, it was noted in which trap, if any, the spiders were caught. Each trap pairing was tested at least 50 times. Only spiders that did not choose a trap during their first experiment were used again. The experimental comparisons were performed in a laboratory setting to cut down on external stimuli that may influence trap choice, such as odors, air currents, temperature, etc.

**Table 1.**
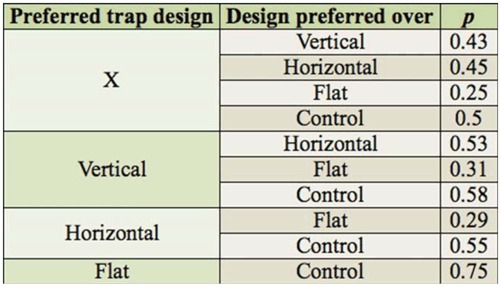
Estimated preference probabilities obtained from the fitted model.

**Table 2.**
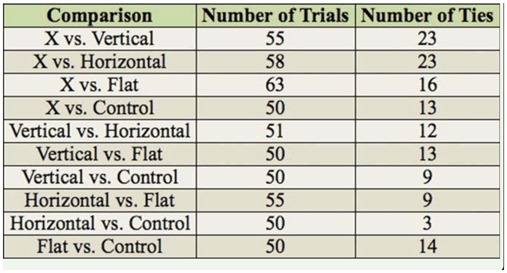
Number of trials and ties.

### Statistical analysis

A Bradley-Terry model was fitted for paired comparisons in SAS© 9.2 (www.sas.com) with PROC LOGISTIC and PROC GENMOD, where ties (spider prefers neither trap) are removed. The Deviance and Pearson Goodness-of-Fit Statistics in PROC LOGISTIC yield *p*-values of 0.09 and 0.10 respectively, the Hosmer-Lemeshow *p*-value is 0.21, and the Lagrange Multiplier Statistic for non-intercept in PROC GENMOD yields a *p*-value of 0.03, which suggests that there may be a problem with the fit of the Bradley-Terry model.

## Results and Discussion

The estimated preference probabilities obtained from the fitted model are listed in Table 1. The probabilities suggest the following ordering of the five traps for catching *L. reclusa* (least preferable to most preferable): Control < X trap < horizontal bar trap < vertical strut trap < flat trap.

In addition to the possible problem with the model mentioned above, there was a fairly high percentage of ties in the data set (Table 2). As a result, an extended Bradley-Terry analysis that adjusted for ties was implemented in SAS. Here, a tie was interpreted to mean that each trap receives one half of a choice. For example, assume that 50 trials were performed for a pair of traps, and the first trap was chosen 23 times, the second trap was chosen 22 times, and neither trap was chosen 5 times. In the adjustment for ties, pseudo-data were generated, where the first and second traps were chosen 25.5 and 24.5 times, respectively. Turner and Firth (2012) find that this simple and intuitive approach to handling ties works well in practice and generally yields results very similar to those obtained from much more sophisticated analyses, which have the disadvantage of being much harder to implement and interpret.

A Bradley-Terry model for paired comparisons was fit with the pseudo-data values in SAS. The Deviance and Pearson Goodness-of-Fit Statistics in PROC LOGISTIC yielded *p*-values of 0.17 and 0.18 respectively, the Hosmer-Lemeshow *p*-value was 0.35, and the Lagrange Multiplier Statistic for non-intercept in PROC GENMOD yielded a *p*-value of 0.06. Obtaining insignificant *p*-values for each of the four goodness-of-fit procedures suggests that the extended Bradley-Terry model fits the data well.

**Table 3.**
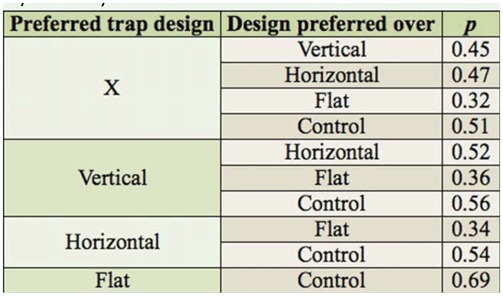
Estimated preference probabilities obtained from the adjusted analysis.

**Table 4.**
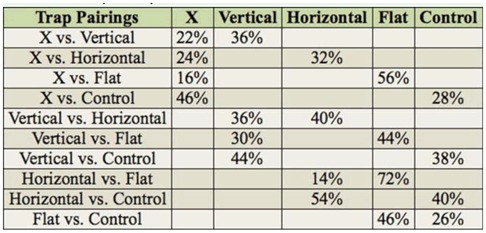
Trap comparisons.

The estimated preference probabilities obtained from the adjusted analysis are listed in Table 3. The probabilities yielded the following ordering of the five traps for catching *L. reclusa* (least preferable to most preferable): Control < × trap < horizontal bar trap < vertical strut trap < flat trap.

In summary, analyses that exclude ties and analyses that included ties agreed on the same ordering of the traps.

The flat trap was chosen more than the other traps in the pairwise comparisons (Table 4). However, the flat trap was the least user-friendly trap of those tested, since there was no barrier to prevent accidental glue contact from non-arthropod victims such as children, pets, etc. The other traps had some type of cardboard “roof over the glue part, serving as a physical deterrent for unwary or inquisitive animals and/or children. The standard, unmodified control trap design performed poorly against all of the modified designs, even though it had a much larger glue perimeter (55.88 cm) and glue surface area. Exposed glue perimeters for the X, all vertical, vertical with horizontal bar, and flat traps were 18.42, 17.78, 19.69, and 36.83 cm, respectively. Perimeter comparisons can yield only a partial explanation for the differences in trap selection, because the flat trap had 53% more exposed perimeter than the other modified traps, yet it was chosen 14% more often than the horizontal bar trap design. It also outperformed the control trap, which had 66% more exposed perimeter than the flat trap. Also, the cardboard backs and struts on the other 3 modified traps may have facilitated spider escape, as there was no glue on those areas. The experimental roofed traps were constructed of chipboard cardboard, a different material than the commercial roofed traps, so the different results obtained with the experimental traps vs. the commercial traps cannot be ascribed solely to different design shapes. It is possible that external stimuli detectable by the spiders may have affected results. A lid over the experimental chamber could reduce possible external environmental stimuli, such as air currents, temperature and humidity fluctuations, light intensity, etc. Additional study with more trap designs may allow further optimization of the brown recluse trap design.
